# Piezoelectric Behaviour in Biodegradable Carrageenan and Iron (III) Oxide Based Sensor

**DOI:** 10.3390/s24144622

**Published:** 2024-07-17

**Authors:** Vytautas Bučinskas, Dainius Udris, Andrius Dzedzickis, Jūratė Jolanta Petronienė

**Affiliations:** 1 Department of Mechatronics, Robotics and Digital Manufacturing, Vilnius Gediminas Technical University, LT-10105 Vilnius, Lithuania; andrius.dzedzickis@vilniustech.lt (A.D.); jurate-jolanta.petroniene@vilniustech.lt (J.J.P.); 2 Department of Electrical Engineering, Vilnius Gediminas Technical University, LT-10105 Vilnius, Lithuania

**Keywords:** force sensor, dynamic properties, k-carrageenan, proton transfer, piezoelectricity

## Abstract

This paper is dedicated to the research of phenomena noticed during tests of biodegradable carrageenan-based force and pressure sensors. Peculiar voltage characteristics were noticed during the impact tests. Therefore, the sensors’ responses to impact were researched more thoroughly, defining time-dependent sensor output signals from calibrated energy impact. The research was performed using experimental methods when a free-falling steel ball impacted the sensor material to create relatively definable impact energy. The sensor’s output signal, which is analogue voltage, was registered using an oscilloscope and transmitted to the PC for further analysis. The obtained results showed a very interesting outcome, where the sensor, which was intended to be piezoresistive, demonstrated a combination of behaviour typical for galvanic cells and piezoelectric material. It provides a stable DC output that is sensitive to the applied statical pressure, and in case of a sudden impact, like a hit, it demonstrates piezoelectric behaviour with some particular effects, which are described in the paper as proton transfer in the sensor-sensitive material. Such phenomena and sensor design are a matter of further development and research.

## 1. Introduction

The registration and accurate measurement of the local force impact applied to the human body or any material with a relatively low Young’s modulus and high failure strain is a challenge. The reasons are multiaxial deformation, complex force distribution, and uneven deformation of the register sensor. Currently, a limited quantity of articles provide comprehensive research on the accuracy of force or pressure sensors due to the influence of uneven deformation and applied force distribution in soft, flexible materials [1,2,3,4,5,6]. Respectively, most developed resistive, capacitive, piezoresistive, or optical tactile sensors are intended to operate at low forces and low deformation levels. Tactile sensors that exhibit significant deformation are not expected to be fast and accurate. Therefore, the development and research of soft-sensitive materials for force or pressure impact measurements is required.

Impact force measurement is relevant in various fields, especially biomechanics, sports training, and boxing. A maximum impact force of about 4 kN of boxer hit on the test dummy result was reported in [7,8]. The Yoshikazu Tanaka group [8] provided a successful impact force sensor and evaluated operating forces influenced by bending deformation, a shear force caused by friction, the Poisson effect of the contact material, and the lateral compressive force caused by the deformation of the flexible material.

Determining the impact of the strength of the force is relevant for structural health monitoring and safety assessment. Liu et al. [9] investigate the uncertainly orientated impact force identification system for composite structures considering signal noise, material dispersion, and manufacturing errors. 

Porous materials are well-known as protective materials used to ensure the safety of humans and structures in industry. Flexoelectric porous material, for example, porous polydimethylsiloxane, is mentioned as a sensitive material suitable for the detection of impact force sensing [10]. 

A convenient piezoelectric sensing mechanism that generates a polarised electric charge under deformation inside the material is attractive for the development of shock force sensors because polarized charge increases in direct proportion to external force or pressure [11]. However, piezoelectric materials are also sensitive to the direction of applied force and stress distribution on their surface.

The properties of composite membranes depend on the compatibility of material and dispersion of components in the polymer matrix [12]. During the preparation of polymer composites, the sedimentation, aggregation, and heterogenous distribution in the polymer matrix are encountered [12]. 

Compositions of natural biopolymers and various electrically conductive materials, such as carrageenan and iron (III) oxide composition, presented in our previous reports [6,7] demonstrate properties suitable for force impact sensing. On the other hand, the composition of materials with different electrical charge transfer mechanisms results in a high sensitivity to the concentrations of all composition components and complex electrical properties. For example, k-carrageenan with high proton conductivity [13,14,15], in which protons migrate through a hydrogen-bonded network due to the hydrophilic nature of this biopolymer, impacts the electrical properties of iron oxide, which is typical for semiconductors.

This research provides the electrical response of the experimental sensor to the dynamical impact of the phenomena obtained on the newly discovered material mixture. Demonstrated sensitive material properties open a comprehensive perspective for force sensor development, given as experimental graphs, and will launch the start of a series of papers on further research. 

## 2. State of the Art 

### 2.1. Carrageenan as Multifunctional Biopolymer

In recent years, biopolymer-based hydrogels have attracted increasing attention because of their suitability for various applications. A three-dimensional network of natural polymers can hold large amounts of water molecules. Natural hydrogels are attractive for different industries and biomedical purposes due to their inherent properties such as biocompatibility, cytocompatibility, biodegradability, non-toxicity, and semi-solid state [16]. Hydrogels are generally hydrophilic solid materials because assembled molecular networks can capture large amounts of water molecules without dissolving or losing integrity [17,18]. Chemical bonds cross-link these biopolymer hydrogels: covalent bonds, ionic interactions, hydrogen bonding, as well as physical interactions, such as coordination, electrostatic forces, hydrophobic and dipole-dipole interactions, or the entanglement of the molecule chain between segments of the polymer network [19]. The cross-linking nature within the biopolymer frame determined the hydrogel nature. It could be chemical or physical. In chemical hydrogels, the molecular chains of the scaffold are covalently cross-linked, making robust and irreversible hydrogel. Reagents used for cross-linking procedures may have harmful properties and must be removed before using a hydrogel. Physical gels are formed when chains of polymer building blocks are held together by noncovalent bonding but by interactions such as hydrogen bonding, hydrophobic, aromatic π-π bonding, or electrostatic [20,21,22,23,24]. Natural-origin polymer hydrogels, as usual, have advantages due to non-toxicity, biodegradability, and renewal of resources [25,26].

However, hydrogels made from natural polymers suffer from weak mechanical strength, high molecular variability, and immunogenicity compared to synthetic polymers with stable and predictable polymer architecture, mechanical properties, and long-term stability [27]. Naturally derived polymers, such as cellulose, chitosan, starch, pectin, gelatin, agar, carrageenan, and alginate, have excellent application in the food industry in different stages of application, from consumption to packing material. Among various types of electroconductive materials and electrolytes, polymer-electrolytes are the best materials in solid state technology due to their flexibility, compatibility, low weight, and safety to use [28].

Carrageenan is one of the most widely used natural polysaccharides, and it caught the attention of our scientific group due to its high molecular weight. Characteristics of water-soluble polysaccharides obtained from different types of algae depend on the species of water plants from which it was extracted. The most common carrageenan (CG) types are iota, kappa, and lambda, and the number of ester sulfate groups categorises them. The difference in the number of functional groups results in different properties of CG, including solubility in water, flexibility of the prepared sample, physical strength, and others [29]. Kappa(k)-CG has the lowest amount of sulfate ester groups but has better film-forming abilities than other kinds of carrageenan. The mechanical properties and water–gas barrier are limited. Improving the mechanical properties, vapor permeability, and antibacterial activity of k-CG are among the main research problems that limit the broad application of this cheap and renewable resource of nature [30]. Carrageenan has many desired biological properties: antioxidant, anti-ageing, antiphotoaging, anti-melanogenesis, and photoprotective for human skin effects [31,32,33,34,35,36]. The k-Carrageenan unique double helix structure can enhance the elasticity of the material [37]. This material has worked well as an antiviral in the fight against COVID-19 [38,39,40]. Due to the safe contact of this polysaccharide with human skin and its efficacy in reducing intracellular reactive oxygen species induced by ultra-violet B light (range 290 to 341 nm), or UVB, to normal human keratinocytes, carrageenan can be applied to skincare or treatment products [41,42] and as a successful viscosifier and binder in toothpaste [43], or the food [44] of food packages [45,46]. Careful biomedical analysis of the effect of carrageenan in contact with living organisms is an integral part of the development of force sensors due to the possible influence of contact with skin [47,48]. The correlation between the morphology of hydrogels and their mechanical properties was demonstrated in changes in their rheological properties [49]. 

Different methods appropriate for specific goals can be employed to investigate the characteristics of prepared biofilms. Moisture content, water contact angle, water vapour permeability, the morphology of film, and mechanical properties such as tensile are essential for hydrogel characterisation [50]. Combinations with synthetic polymers help to create films that reduce the disadvantages of the natural hydrogel. This is especially useful when the synthetic polymer increases valuable properties and has insignificant toxicity. PVA is one of the most widely applied synthetic polymers combined with natural carrageenan to produce a hydrogel for biomedical applications [16,51,52]. The wide applications of carrageenan in the food industry have also been carefully evaluated due to possible adverse effects on the human organism [53,54,55,56]. Therefore, by evaluating the environmental friendliness of carrageenans, the potential risks and sustainability needs of this material, the benefits of renewable resources, and the challenges posed by the molecular diversity arising from changing environmental conditions, it is an excellent all-purpose form of natural hydrogel. 

As a global issue, the emergence of antibiotic-resistant strains of bacteria poses a significant threat to public health. This problem has led scientists to search for and develop new antimicrobial materials and carefully review the relevant properties of already known materials [57]. CG received a great deal of attention during the last pandemic due to its antiviral properties. These properties affected the use of carrageenan in various sensing applications, especially in wearable and tactile sensors, because of the promising antibacterial properties of this polysaccharide. 

### 2.2. Iron (III) Oxide Influence on Electric Conductivity

The metal oxides and conducting polymers act as pseudo-capacitors and electric double-layer capacitors. Pseudo-capacitors occur due to Faradaic reactions between the electrolyte and the surface of the electroactive electrode material [58]. Composites based on conductive polymers and metal oxides are developed to increase the specific area of metal oxide, preventing the agglomeration of oxide particles, expanding the voltage window, and resulting in mechanical stability. The composition of iron oxide and conductive polymers results in electrically conductive materials exhibiting various effects and phenomena. Generally, iron oxide exists in various crystallographic forms based on the atomic arrangements of iron and oxygen ions. The most stable form of iron oxide crystal is a corundum-like structure. The α-Fe_2_O_3_ has poor electrical conductivity (10^−14^ S/cm), influencing the resistance-to-charge transfer between electrolyte and electrode [58]. In addition, the α-Fe_2_O_3_ capacity of Fe_2_O_3_ is decreasing due to the increased volume during several charge–discharge cycles [58]. 

Alpha-Fe_2_O_3_ is a cheap and abundant semiconductor. It has a visible light indirect (phonon-assisted) band gap of 2.06 eV (600 nm) due to a d–d transition and a direct band gap at 3.3 eV (375 nm), associated with the ligand-to-metal charge transfer process [59]. The main limiting factor of the photoelectron conversion capabilities of α-Fe_2_O_3_ is poor charge transfer efficiency and the presence of surface trapping states [58,60,61].

The iron oxide clusters are monomeric and dimeric, the basic units of minerals based on iron oxide based minerals [62]. Iron oxide, especially in the form of nanoparticles, exhibits suitable properties as a candidate for interaction with bacteria and demonstrating antimicrobial efficiency [57].

Iron oxide also has a wide range of magnetic properties, and because of its versatility, a wide range of applications are available. In addition, superparamagnetism at ambient temperature of nanoparticles with fluctuation of the magnetic moment in a single domain is another interesting physical parameter [63,64,65,66]. The photothermal effect of Fe_3_O_4_ with the various shapes of magnetic nanoparticles has wide application in cancer therapy [63,67,68]. In [65], numerous reports of successfully synthesised different α-Fe_2_O_3_ structures are analysed and proposed for new application fields, claiming that the magnetisation levels of new structures resulted from the increased number of spins caused by the breaking of the bonds of surface atoms. Therefore, the magnetic properties depend on the size, particle shape, and microstructure [69,70,71,72,73]. Structural disorder within the particles or on the surface greatly influences the magnetic properties of particles, especially in nanoparticles of the same size [69]. In the synthesizing of iron oxide particles, various properties can be controlled by the choice of synthesis method, coating techniques, and sample preparation technology. Crystal structure impurities play an important role in particles’ magnetic behaviour. Recent publications show that efforts are focused on developing simple, less costly methods and ensuring compatibility with living systems [74]. 

Iron oxide was represented as a promising negative electrode for supercapacitors due to its wide operating potential, high redox activity, low cost, and environmentally friendly properties [58]. 

Furthermore, the conductivity of Fe_2_O_3_ is not the same in different directions of the crystal lattice, is evaluated as the valence change in iron cations, and helps to calculate the mobility of localised electrons in a single particle [75]. The high value of electronic coupling indicates that charge transport in hematite is adiabatic. However, the magnitude of the charge transfer rate depends on the energy of electronic coupling and reorganisation energy [75]. Investigation of iron oxide surfaces by low-energy electron diffraction shows the nonstoichiometry of the surface. The study presented in [76] contributes to a better understanding of how specific sites on mineral surfaces interact with surrounding media to alter interfacial electron transfer kinetics.

The heterogeneity of the electron states in transition-metal oxides is observed in nanocrystals, and chemically synthesised iron oxide properties depend on their size. The investigation results presented in [77] help to understand the relationship between d–d transition, magnetic pairing, and charge transfer in transition metal oxides. Thus, the smaller particles have more diversity in their properties than the bulk system due to the quantum size and finite size effects, which also show a strong dependence on structure.

### 2.3. Effect of Friction and Other Tribological Issues 

The nature of the interfacial interaction potential is known, as well as the tribological properties of the materials. Mechanical interaction between sliding contact surfaces is the main property causing surface friction. There is a relationship between friction coefficients and ionic potentials of various oxides. 

In tribological situations where a few dissimilar solid bodies may rub against each other, sliding surfaces are covered by more than one kind of oxide. Crystal chemistry can be applied to evaluate the physical parameters [78]. The vibration spectrum of surface-surface for soft surfaces without applied load is influenced by coupling between intermolecular and surface vibration. This coupling is very important for stiff surfaces when the applied force is increasing. 

The friction forces for soft and stiff surfaces are similar when the load is low, but when the applied load is significant, the stiff surface exhibits higher friction forces. This fact explains the dependence between surface–surface vibrational spectra for soft and stiff surfaces concerning the applied load. During the load increase, the soft and stiff systems enter the energy wells, requiring additional energy to overcome the potential energy barriers to slide and increase friction. When the sliding rate decreases, there is a necessary time for structural relaxation and energy transfer between surfaces.

Knowledge of the optimised potential along the sliding direction (analogous to the reaction path potential) can help to understand the tribological behaviour under loading and interfacial sliding [79]. With low sliding, the system can capture a large fraction of the local intermolecular potential energy surface, and the friction reaches maximum. Therefore, the friction forces encountered at low slide velocities are much higher when sliding velocity is high [80]. The relaxation rate of the vibration energy from the surface to the surface interface during sliding depends on the intermolecular vibration frequencies on the interface [81]. Sliding friction forces are similar for soft and hard alumina surfaces at low loads. However, the friction forces on the rigid surface are more significant with higher load. Increasing the load forces the rigid surfaces into deeper interfacial potential energy wells, resulting in higher sliding barriers and frictional forces [79].

### 2.4. Proton Type Conducting Biopolymer Coupling with N-Type Semiconductor Oxide

Iron oxide is known to be a poor semiconductor with high resistivity. Due to vacancies in the valence band and a conduction band populated by electrons, Fe_2_O_3_ is working as an n-type semiconductor [82,83]. The M. Ishii group claims that electron transfer reactions between semiconductor and dopant molecules are essential for the chemical doping of molecular semiconductors and for electron transfer reactions [84]. Ion implantation is a common method in semiconductor manufacturing processes. Looking for analogous or similar solutions, according to the J.N. Klug group [85], proton implantation has limits. Semiconductor ionic materials attract researchers because of their structural and chemical properties, which make them suitable for efficient proton transport. They are a new class for fuel-to-electricity conversion [86,87]. Understanding the surface or interfacial conductance of proton transport in oxides can lead to the development of future materials [87]. There are a few theories of proton transfer: Grotthus, Vehicle mechanism in liquids [88], and proton transportation in perovskite-type oxides were studied by many scientific groups [87]. The surface and interphase proton transport phenomena differ from bulk transportation mechanisms [87]. Therefore, mesoporous α-Fe_2_O_3_ ceramic membranes are characterised as a proton conductive material [89].

## 3. Materials and Methods

All tested samples were made using deionised water prepared in the laboratory. All chemicals used (isopropanol, carrageenan, glycerol) were purchased from Sigma-Aldrich (Taufkirchen, Germany); only iron oxide was purchased from Labochema (Vilnius, Lithuania). All materials for the experiment were obtained according to the laboratory’s internal procedures. Carrageenan, as the main material for the hydrogel film, was prepared from algae extracted from the sea weed *Furcellaria lubricalis* in the scientific laboratory of Liepaja University according to the procedures described in [31,90,91]. Preliminary analysis with nanoparticles and microparticles, but after collecting the desired results and taking into account the direction of sustainability, cheap iron oxide pigment was chosen for the main experiments. The distribution of iron oxide (III) particle size was evaluated by additional analyses.

Particle size definition. The particle size distribution was defined as statistical values by device software and was submitted as a distribution density curve q_r_(x), the sum of distribution Qr, which shows the standardised total quantity of all particles with q-equivalent diameters or equal to x. The specific surface was calculated to include 0.1 g of iron(III) oxide particles by using Analysette 22 software (version NeXT Micro) for the next step of explaining charge transfer in carrageenan-Fe_2_O_3_ film. The distribution of Fe_2_O_3_ microparticles in the selected product was with one maximum, corresponding to the distribution of naturally grounded and sieved solids. The sum of the distribution shows the total quantity of all particles. The curve of density distribution is the first derivative of all particles. After evaluating the data according to the distribution of particle size, the colloidal solid electrolyte was prepared.

Sample preparation. Cleaned and treated with isopropanol, the mass carrageenan biopolymer was mixed with glycerol and iron oxide powder in certain proportions. The electrical conductivity of the prepared carrageenan liquid after the cross-linking procedure was 0.27 ± 2 mS/cm. The resistance of this cross-linked carrageenan was 0.038 ± 1 MΩ. Before the drying procedure, the prepared sample, with other materials, including the sensor film (iron oxide, plasticizer) with water residues, has a resistance of about 0.090 ± 5 MΩ. Resistance values depend on the amount of iron oxide in the final product. Dried film samples measured surface resistance depends on the amount of plasticizer and water amount in this hydrogel and varies between 1.2 MΩ and 13 MΩ, not measurable values when the sample completely loses moisture. An interesting fact about this carrageenan film is its ability to restore the conductivity function when exposed to a wet environment if it is unlaminated or immersed in distillate water for a short moment. The mechanical and electrical properties desired for this investigation depend on the proportions of the selected components. According to our previous investigation [91], we selected the film composition as follows: 3 g of carrageenan biopolymer diluted in 100 g of H_2_O, with 18 g of Fe_2_O_3_, and 18 g of glycerol. The prepared film after the drying procedure was of 2 mm thickness, flexible, and maintaining the given shape. Alumina wires were attached to the surfaces of the prepared CG sample on different sides of the film. After the electrodes were attached to the film, all this system was wrapped into parafilm for a few reasons: to give some integrity without affecting the elasticity of the main material and to be sure that the conductor’s surface area remained the same during all experiments.

Devices. For the iron oxide particles, a distribution analysis was performed in suspension using Analysette 22 MicroTec-plus, from Fritsch GmbH, Weimar, Germany. The Hanna Dist 4 (mS/cm) tester from Hanna Instruments, Woonsocket, RI, USA was applied for electrical conductivity measurement. The resistance of this liquid was measured by multimeter UNI-T UT55.

For potential measurements, the Hantek DSO 6082BE digital oscilloscope, from the Netherlands, was applied, with two independent channels (bandwidth 80 MHz), and a sampling rate up to 250 MS/s. The measurement results are saved in a CSV file, with 10,240 samples for each screen, consisting of 10 divisions on the time axis. The value of the time unit used for the measurements ranged from 2 to 20 ms. In the results shown, the voltage step is 100 mV. The measurements are made in single pulse mode with a threshold trigger value of 16.3 mV. The oscilloscope was preferred due to rapid voltage change registration during a short time of performance.

**Figure 1:**
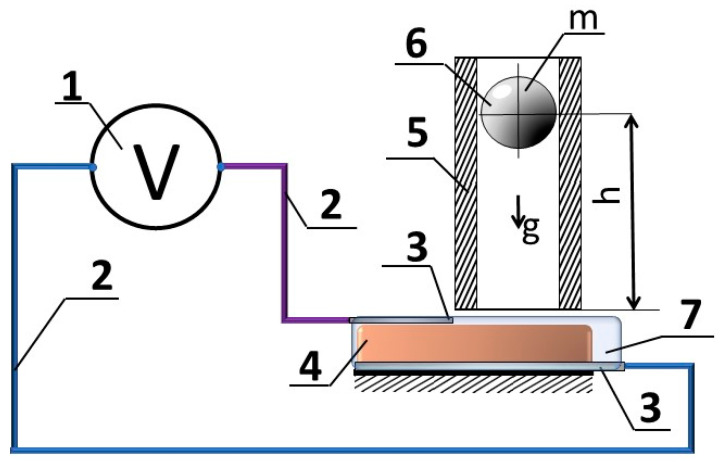
Visual representation of experiment: **1**—oscilloscope as voltage registering device; **2**—connecting wires; **3**—electrodes; **4**—CG film with Fe_2_O_3_; **5**—plastic tube of selected height: 0.15 m, 0.3 m, 0.6 m, 0.9 m; **6**—stainless steel ball: 0.007 kg; **7**—lamination.

Methodology. The experimental setup, presented in Figure 1, was used for the sensor mock-up. The impact to the sensor was normalised by using a steel ball free-fall from the prescribed height; that gives a defined energy of impact. The energy of the ball is simply defined using Formula (1):

(1) $$E_{p}=mgh;$$

where *m*—steel ball mass, *g*—acceleration of gravity, *h*—height of free fall of the ball.

According to energy conservation law, kinetic energy at the time of impact is equal to the potential one, which defines the energy of impact:

(2) $$E_{k}=E_{p}=\frac{mv^{2}}{2};$$

where v denotes the speed of the ball.

Keeping the same conditions of ball impact, it is proportional to the height of the ball free-fall, which makes the sensor response relatively accountable.

While the aim of this research is to demonstrate an effect in the sensor material, relative impact evaluation looks reasonable, but in forthcoming tests, the amount of impact will be quantitatively evaluated.

During the test procedure, the drop of the ball from the defined height (0.15 m, 0.3 m, 0.6 m, 0.9 m) and voltage measurements were performed three times per case. A consecutive series of hit effects were observed in the same area of the film. The next test was performed with the other segment of film made previously. The plastic tube was selected based on the diameter of the ball. During the experiment, the ball fell smoothly and hit the inner wall of the tube occasionally; therefore, it can be neglected to the ball energy balance and, as a result, the energy of the ball hit. The principal scheme of the experiment is presented in Figure 1. The bench for conducting an experiment is simple: it is a sample with electrodes connected by wires to the device for voltage measurement. The ball drop using a tube is performed to ensure controlled impact location and sample fatigue level determination. Electrodes are attached to the active sensor film from different sides to ensure the electrical current path through the surface and to enable electrochemical processes inside the sample.

The prepared CG film with Fe_2_O_3_ and glycerol was cut into 30 × 30 mm pieces, mounted in a transparent flat plastic holder, connected by Alumina electrodes of 10 × 50 × 0.05 mm size. The sensor sample was tested to the influence of impact.

**Figure 2:**
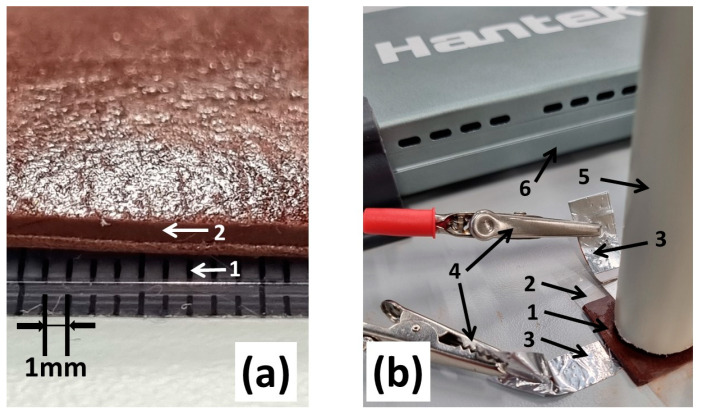
Visual representation of carrageenan film prepared for applied force measurements. (**a**) carrageenan/ iron oxide film cross-section before experiment: 1—ruler; 2—carrageenan/iron oxide film. (**b**) Mounting of sample for the applied force experiment: 1—carrageenan film; 2—lamination by parafilm; 3—aluminium electrodes 10 × 25 × 1 mm; 4—connectors of the oscilloscope; 5—guiding tube for falling ball; 6—Hantek DSO 6082BE digital oscilloscope.

As shown in Figure 2a, the hydrogel sample surface is dry, flexible, soft, and maintains its structure well. Above the hydrogel sample, the tube was installed for ball dropping as shown in Figure 2b. The hydrogel surface relief formed naturally during drying and visible surface defects did not affect the quality of the experiment.

The investigation of applied force vs. distance was realised in this order: the same sample was affected by a falling ball, which was the ball was dropped from different heights onto the film using a tube of suitable diameter as shown in Figure 2b. The time intervals during which the experiment was performed were of 30 s. The time intervals were chosen to be short compared to the recovery time of the film after the applied force. The application of a falling ball instead of another object with an easily calculated area was also chosen to observe how the film reacts to the impact not only on the surface but also in deeper layers.

## 4. Results

### 4.1. Evaluation of Iron (III) Oxide Particle Size

The distribution of the size of Fe_2_O_3_ microparticles used in tested samples corresponds to the normal or so-called Gaussian distribution, which is typical for the powder of naturally ground and sieved solids powder. About 8.16% of the particles analysed had a size of 1.07 µm, about 60% of particles were between 0.5 µm and 1.74 µm, the size of about 30% of the particles varied from 0.5 µm to 5 µm, and about 1% of the particles were smaller than 0.1 µm. After the data were determined according to the distribution of particle size, a colloidal solid electrolyte was prepared using protonic charge transfer of carrageenan gel coupled with semiconductor charge transfer of iron oxide (III).

The curve of the sum of distribution shows the total quantity of all particles in this investigation. The density distribution is the first derivative of all particles. After the data were collected according to the particle size distribution, the colloidal solid electrolyte was prepared. According to the results, the particles of iron (III) oxide in our work act as colloidal material, so the distribution of material properties is less specific in comparison to that of applied nanoparticles.

### 4.2. Sensor Response to the Impact

The results obtained on the sensor’s reaction to the weighted impact are presented below in the form of graphs in the time domain.

**Figure 3:**
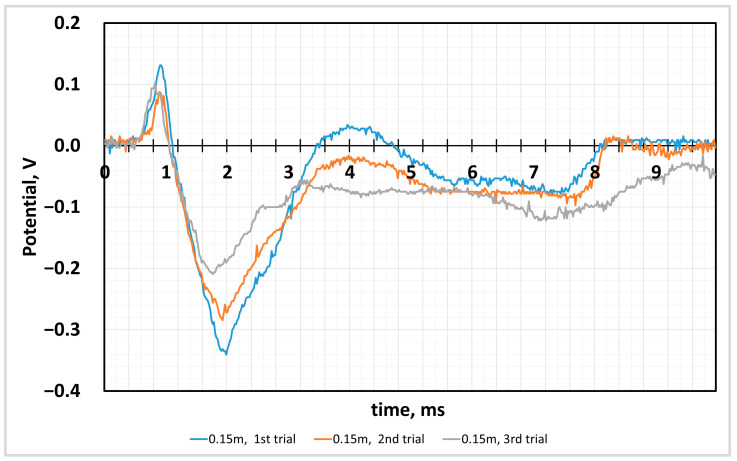
Voltage of the sensor output from mechanical impact. Impact generated by a 10 mm, 7.04 g steel ball from 0.15 m height.

Figure 3 shows the reaction of the CG film to the impact of the metal ball. The experiment was carried out in a series of 120 s between the curve registration and the film location, so the hit was carried out in the same area of the film. The first increase in potential corresponds to the first impact of the applied force, when the ball dropped from a height of 0.15 m. Since the applied force shocks occurred sequentially, fatigue induced in the negative values range was observed as a decrease in potential. The potential change during impact is recorded in the range of positive potential values. Next, we register the potential growth caused by the reaction of the film material. The potential values were recorded in the negative potential section. As we can see from the curves, the rebound of the ball from the film surface is registered at 8 s on the x-axis, which caused a potential change in the range of negative potential values. This change in charge values can be explained by processes that occur due to the nature of the material [92]. The experiment was repeated three times with samples cut from the main film sample to maintain uniform film production.

**Figure 4:**
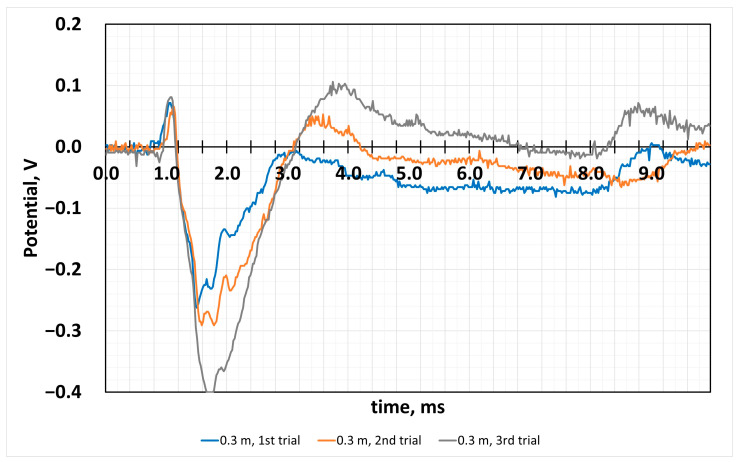
Measured potential dependence on time, when the distance of the falling ball was 0.3 m from the surface. Measurement of applied force on CG film.

The distance of the 0.3 m of falling ball (Figure 4) resulted in a similar value at the first contact moment, and it is about 0.1 V, but the next peak shows that the reaction of the film can be attributed to the physicochemical nature of the proton-transferring CG and semiconductor iron oxide. Fatigue causes a different response than at shorter distances, and this raises additional questions to explain this effect. As we can see from this experiment, the potential values of the film reaction to the hit differ compared to the distance to the drop ball, by two times less. The potential value in the negative potential values reached −0.4 V in the third curve, and after the values decreased, this can be attributed to the fatigue of the sample. However, the potential increases later to positive potential values, when the total numerical value from the most negative to the most positive reaches almost 0.45 V, which is plenty efficient for 1 mm thick film.

**Figure 5:**
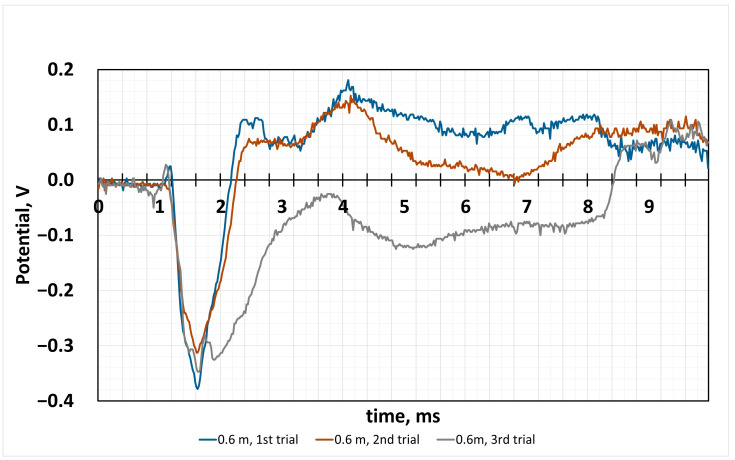
Measured potential dependence on time, when the distance of the falling ball was 0.6 m. Measurement of applied force on CG film.

When the ball is thrown from 4 times the initial height (Figure 5), we observe the following changes in potential: at the impact moment, the positive potential of about 0.05 V is registered, and later, the potential increases in the negative values and reaches −0.38 V. After the ball bounces further, the values in the positive range of potential reach 0.18 V, and the initial impact is already higher.

This experiment also raises a number of questions about the sharp potential growth caused by the film reaction from the ball bouncing off the surface. This can only be explained by the activation of additional reactions inside the film, because it does not correspond to the expected fatigue-induced reaction. Because proton charge transfer in all electrolytes is similar but much slower than electron transfer, it would be possible to analyse which of the substances is the most limiting, carrageenan or iron oxide. The maximum value difference was of 0.4 V.

**Figure 6:**
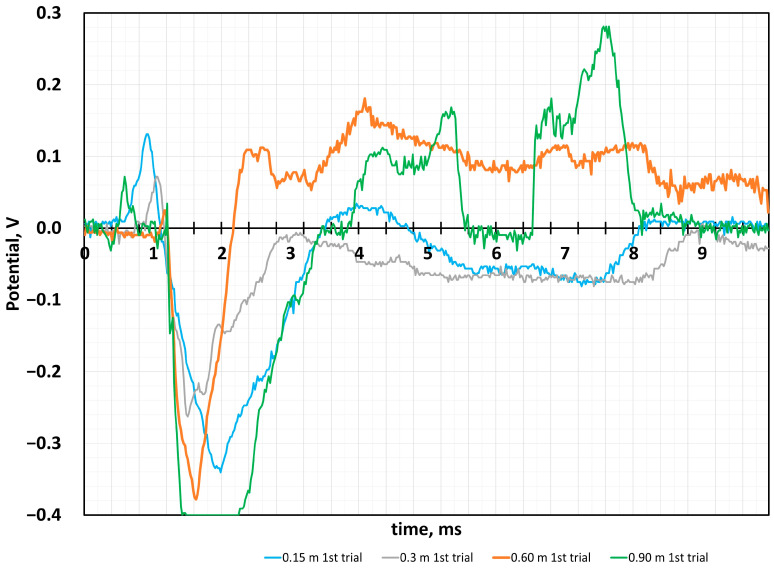
Measured potential dependence on time in comparing of applied distance of falling ball, results of first hit experiment.

The data obtained from all of the experiments presented in Figure 6 show that the shock response of this film is very complicated according to the recorded signal potential values. The effect of the ball bouncing off the surface is obvious and causes a chain-like reaction to the applied force. When comparing all the experiment data, the following change in potential values was observed. The 0.9 m ball-falling height corresponds to the highest voltage jump; a more significant voltage fluctuation shows the ball’s bouncing after reaching the sample surface. Such a response shows sensor performance and its ability to sense fast repeating hits (3 ms). The overall sensitivity of the research material evaluating force impact according to Formula 1 is equal to 0.86 J/V.

Discussing the obtained results in more detail, we must note that the initial measured potential change is only 0.07 V at the moment of ball contact with the mechanically sensitive film. Then, with a delay of 0.5 ms, the increase in potential towards negative potential values was registered. Thus, after a certain time after impact, the carrageenan force sensor generates an electrical signal greater than the initial value, and it is more than 0.4 V. These potential differences, registered later than the physical impact to the film was performed, can be attributed to processes that occurred inside the film. The discussion of the possible events during the impact on the film based on knowledge of material science and electrochemistry is in the text below.

**Figure 7:**
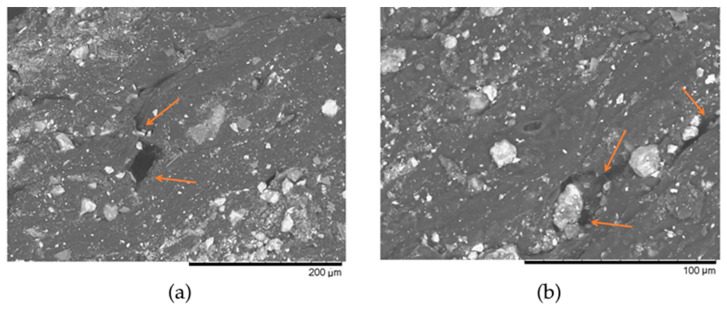
SEM micrograph of CG film with iron (III) oxide particles. Size = 1280 × 1100; (**a**) DPI = 182.65; Conditions: V_acc_ = 15.0 kV; Mag- = ×400; Pixel size = 347.66 (left). (**b**) Mag- = ×1000; DPI = 182.65; Pixel size = 139.06 (right).

In comparing all applied heights of the falling ball (Figure 6), we can claim the hit of the ball causes processes inside the film of different natures of charge transfer. At the first contact of the ball with the film, the enlarging positive potential is noticeable, which is attributed to the mechanical approach of iron (III) oxide particles or to semiconductor charge transferring. After the hit, we register the potential growth in negative scale values. This process is a complex of carrageenan sulfate groups working to transfer protons because of the change in the surface area around the iron oxide particle. We want to explain this process by the cavities near the iron oxide particles, which contain the residual solution that acts as a liquid electrolyte (Figure 7). This idea of ours should be confirmed by the measurements obtained at the maximum distance at which the bounce of the ball from the carrageenan film was recorded. In the test marked with the green line, we had two ball bounces of the surface. In this part of the curve, we see potential changes only in the zone of positive potential values. In the zone of negative potential values, the changes are insignificant. Such measured values can be explained by the different charge transport nature of the materials we selected for our force sensor film. As mentioned above, the carrageenan biopolymer has a sulfate group that can release a hydrogen cation during compression. We found this in practical experiments when the film produced did not contain iron oxide particles. This charge transfer is guaranteed by the water molecules in the gel as well as water in the form of droplets, which remain because of the wetting properties of the iron oxide surface when hydrophilic and hydrophobic substances have different adhesion to water molecules.

In this SEM image, we illustrate the distribution of free water droplets not incorporated into the biopolymer structure within the film structure. These droplets play a very important role in the performance of the film because they are involved not only in the compression or impact of the film, but also in ensuring the stability of the water content, which is also very important in maintaining the quality of the film when it is not laminated. Thus, in this hydrogel film, the materials interacting in the three-phase junction ensure the charge transfer by regrouping during impact or compression.

**Figure 8:**
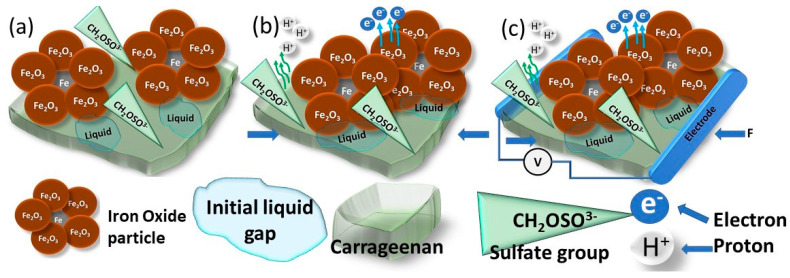
Visual representation of charge transfer in carrageenan film generated by applied force. (**a**) Visual representation of carrageenan film with iron (III) oxide particles; (**b**) charge generation in prepared film, the release of charge-carrying particles due to force; (**c**) visual representation of experiment scheme of film under short time mechanical load.

As we can see in the SEM images, the structure of carrageenan film contains iron oxide in trigonal crystalized hydrophilic powder [93,94]. Materials containing iron oxides exhibit sensibility to compression because of a large amount of defects in their structure, such as ion vacancy, and some impurities make them act as a semiconductor [95]. Iron oxide, under mechanical load at ambient pressure, demonstrates measurable electron hopping between iron octahedral sites, resulting in a charge transfer in iron-rich phases [96]. R. Morris [96] declares that electronic exchange between divalent and trivalent iron ions is registered in a compression of less than 7% of volume, and a small amount of Fe^3+^ is responsible for electrons hopping. The electrical resistance of the powder of structured material depends on the porosity, and with increasing pressure, porosity reduces and electrical resistance is lower [97]. Due to the friction between particles, applied pressure can force the descaling of the dielectric layers, when the powder structure is two layers: inside metal and outside metal oxide. These two phenomena enable an effective decrease in resistivity by increasing the pressure in the powder [97]. Thus, the iron oxide powder already provides a good response to the impact of applied force. Based on this information represented in the articles mentioned above in the text, we presented a visual representation of the possible charge generation and charge transfer when a ball hits a carrageenan film. Visual representation of charge generation during mechanical force hit to the surface of carrageenan film is presented in Figure 8. As it was discussed, the cross-linked carrageenan molecules have sulfate groups, which are involved in charge transfer, making the carrageenan the proton conductive material. Another charge-transferring material is iron (oxide) particles. The compression results in iron (III) oxide particles approximation, causing the free electrons to move. At the point of contact between carrageenan film and iron (III) oxide particles (Figure 7) some gaps remain, which are filled with initial solution residues, and this liquid in the gaps participates as another phase in which hydrogen ions or so-called protons are easily transported. Thus, a heterogenous and very complex system composed of several different charge-transferring materials constitutes the proposed force sensor film.

The composition of iron (III) oxide and a carrageenan biopolymer film was developed to act as a force sensor. When the film ball falls on the surface from a height of 0.15 m, a potential change of approximately 0.4 V is recorded. When the ball fell on the film from higher heights, the potential values also increased to 0.4 V. However, if we compare the potential values in the zone of positive potentials, we must notice that the values recorded during the first impact compared to the potential values caused by the bounced ball are significantly lower than those of the mentioned secondary impacts. Thus, in this case, if film fatigue acts as a signal amplifier, then the first hit causes a change of 0.08 V, the second hit—0.16 V and the third hit—0.28 V in the positive values. The application of this kind of signal can be complicated.

The next moment after the ball reached the surface, the values of the potential in the negative zone were registered. The recorded potential change during the hit has the shape of a sinusoid during the milliseconds of the third measurement. The first maximum can be explained by the rearrangement of the film-forming materials during the impact. The behaviour of hydrogel during the hit and distribution through the gel body or structure with solid, more hard inclusions of iron (III) oxide depends on the measured potential. The record of negative potential values may be due to the compression of iron oxide particles [98,99,100] and next peaks in the positive value range were registered because of the compression of carrageenan with electrochemical active sulfate groups. The apparent interactivity to impact can only indicate the reaction rate of complex electrochemical processes in the transport of hydrogen cations from the hydrogel structure through free water droplets toward the surface of iron oxide crystals. Both biopolymer and iron (III) oxide particles are involved in the charge transfer. In these measurements were found the cumulative potential values of two types of charge transfer.

## 5. Discussion and Conclusions

Specific charge distributions and their transfer take place at the junction of phases; in this case, we have a solid phase of iron (III) oxide in contact with the gel and electrolyte droplets interspersed at the junction of these two phases. Specific charge distributions and their transfer takes place at the junction of these phases. In this case, we have a solid phase of iron oxide in contact with the gel and electrolyte droplets interspersed at the junction of these two phases. This complex set of phase junctions with a large real area clearly acts as an efficient charge-transfer system. A collapsing effect is triggered during an external impact. As we can see from practical experiments, the charge transferred from one material to another during the first impact causes a much stronger reaction to the force of repeated but weaker impacts. Our research system of carrageenan film with iron oxide and weak semiconductor properties can act as a shock-responsive sensor. The complex reaction of the film to repeated hits from a bouncing ball is much more intense than the first hit, especially when the height is close to one metre, and is also an attractive feature in sensor development. Obviously, to control the performance of such a composition as a force sensor, the influence of particle size should be evaluated in more detail, but the particles we selected, which are on the borderline between macro particles and colloidal particles in size, were chosen purposefully, so that we could evaluate how useful it is to use iron oxide products of this size for the production without the need for special equipment and a complex process of nanoparticles.

To explain all these obviously working load transfer mechanisms and acting forces, it is possible to carry out other studies that are no longer the object of the science of mechanics.

An equally important place in the production of this sensor was the selection of suitable metals for the production of electrodes. The choice between copper and aluminium, which are the best conductors of electricity, was determined by the chemical inertness of the aluminium surface compared to that of copper, which can oxidise. A possible significant influence of the ongoing processes can be attributed to the traces of isopropanol that remain during the polymerisation of carrageenan, which is a polar compound and is used in the composition of electrolytes.

Overall, considering this biodegradable iron oxide and carrageenan hydrogel as part of a power sensor, we can say that it is a reasonably sensitive product.

## Data Availability

Data are contained within the article.
